# Penile cancer trends and economic burden in the Brazilian public health system

**DOI:** 10.31744/einstein_journal/2020AO5577

**Published:** 2020-10-29

**Authors:** Fernando Korkes, Antônio Flávio Silva Rodrigues, Willy Baccaglini, Frederico Timóteo Silva Cunha, Júlio Slongo, Philippe Spiess, Sidney Glina

**Affiliations:** 1 Faculdade de Medicina do ABC Santo AndréSP Brazil Faculdade de Medicina do ABC, Santo André, SP, Brazil.; 2 University of South Florida Tampa United States University of South Florida, Tampa, United States.; 3 H. Lee Moffitt Cancer Center & Research Institute Tampa United States H. Lee Moffitt Cancer Center & Research Institute, Tampa, United States.

**Keywords:** Penile neoplasms, Costs and cost analysis, Carcinoma, squamous cell, Development indicators, Public health, Unified Health System

## Abstract

**Objective:**

To gather information on penile cancer epidemiologic trends and its economic impact on the Brazilian Public Health System across the last 25 years.

**Methods:**

The Brazilian Public Health System database was used as the primary source of data from January 1992 to December 2017. Mortality and incidence data from the *Instituto Nacional de Câncer José Alencar Gomes da Silva* was collected using the International Classification of Diseases ICD10 C60. Demographic data from the Brazilian population was obtained from the last census by the Brazilian Institute of Geography and Statistics, performed in 2010 and its 2017 review.

**Results:**

There were 9,743 hospital admissions related to penile cancer from 1992 to 2017. There was a reduction (36%) in the absolute number of admissions per year related to penile cancer in 2017, as compared to 1992 (2.7*versus* 1.7 per 100,000; p<0.001). The expenses with admissions related to this condition in this period were US$ 3,002,705.73 (US$ 115,488.68/year). Approximately 38% of the total amount was spent in Northeast Region. In 1992, penile cancer costed US$ 193,502.05 to the public health system, while in 2017, it reduced to US$ 47,078.66 (p<0.02). Penile cancer incidence in 2017 was 0.43/100,000 male Brazilian, with the highest incidence rate found in the Northeast Region. From 1992 to 2017, the mortality rates of penile cancer in Brazil were 0.38/100,000 man, and 0.50/100,000 man in the North Region.

**Conclusion:**

Despite the decrease in admissions, penile cancer still imposes a significant economic and social burden to the Brazilian population and the Public Health System.

## INTRODUCTION

Penile cancer (PC) is a rare disease, accounting for 0.4% to 0.6% of all malignant neoplasms among men in the United States and Europe.^(^^1^^,^^2^^)^ However, it is well documented that the incidence of PC is higher in developing countries, and may account for as high as 10% to 20% of male urogenital tumors in these regions.^(^^3^^,^^4^^)^ Although relatively rare, PC has a 5-year survival rate of approximately 50% (over 85% for patients with negative lymph nodes, and 29% and 40% with positive nodes), and many times results in devastating disfigurement.^(^^5^^)^

In general, PC presents as a palpable, visible and painless lesion on the penis. Nonetheless, patients may complain of pain, discharge, bleeding or foul odor, especially if medical treatment is delayed.^(^^6^^)^ Approximately 95% of PC originate from squamous epithelial cells, and can be categorized as squamous cell carcinoma (SCC) or penile intraepithelial neoplasia.^(^^7^^)^The ability to perform a good physical examination plays a pivotal role in early diagnosis of penile SCC, and circumcision reduces the incidence of tumors.^(^^8^^)^ This hypothesis is exemplified by countries where the medical system and religious practices lead to higher rates of circumcision: Israel, for instance, has the lowest incidence of PC in the world (0.1 case/100,000 men).^(^^9^^,^^10^^)^ On the other hand, men with phimosis present a risk of developing PC of up to 60%.^(^^11^^,^^12^^)^ Ultimately, it is widely recognized that lower socioeconomic status correlates with the occurrence of PC. A population-based study, conducted in Sweden, reported an increased risk of invasive PC among individuals with lower income and level of education. This is probably related to some factors, such as delay in seeking care, disease stigma, fear of treatment, and lack of knowledge about the diagnosis.^(^^13^^,^^14^^)^

In Brazil, PC may account for 2.1% of all neoplasms in men and affects mainly inhabitants of the North and Northeast Regions,^(^^15^^,^^16^^)^ two geographic areas historically marked by great social inequality and extreme poverty. A recent single-center retrospective cohort study showed an age-adjusted incidence rate as high as 6.1/100,000 cases among male inhabitants in the state of Maranhão, suggesting that this northeastern state might represent the highest global PC incidence rate.^(^^17^^)^ Although Brazil stands out among the countries with the highest incidences of PC in the world, there is no current reliable data regarding the economic burden of the disease in the Brazilian Public Health System (SUS – *Sistema Único de Saúde*).

## OBJECTIVE

To gather information on penile cancer epidemiologic trends and its economic impact on the Brazilian Public Health System across the last 25 years.

## METHODS

The Brazilian Public Health System Database (DATASUS – *Departamento de Informôtica do Sistema Único de Saúde Brasil*) represents the main effort of the Federal Government to collect data from the SUS, and it was used as the primary source of data for our study, along with the Hospital Information System (SIH *– Sistema de Informações Hospitalares/Sistema Único de Saúde*) from SUS.^(^^18^^)^ Epidemiologic data were analyzed from January 1992 to December 2017, gathering information on admissions to public hospitals registered under the following codes: penis amputation, oncology penis amputation, and extended total penile amputation. Health care costs related to PC treatment were estimated using the penile amputation code and analyzed according to the annual inflation in US dollars. Unfortunately we were not able to quantify further costs related to preoperative exams, lymphadenectomy, long-term complications or re-admissions. This database includes information from all public hospitals throughout the country, which provide health care to approximately 170 million Brazilians.

Data on mortality and incidence from the Brazilian National Institute of Cancer (INCA – *Instituto Nacional de Câncer José de Alencar Gomes da Silva*) were collected using the International Classification of Diseases (ICD-10) C60.^(^^19^^)^ Demographic data from the Brazilian population during the studied period were obtained from the last census of the Brazilian Institute of Geography and Statistics (IBGE), performed in 2010 and its 2017 review.^(^^20^^)^

Statistical analysis was conducted using (SPSS), version 13.0 (SPSS for Mac OS X, SPSS, Inc., Chicago, Illinois). Groups were compared regarding the differences between 1992 and 2017 using Pearson's χ^2^ test and statistical significance was determined at p<0.05.

## RESULTS

**Figure 1:**
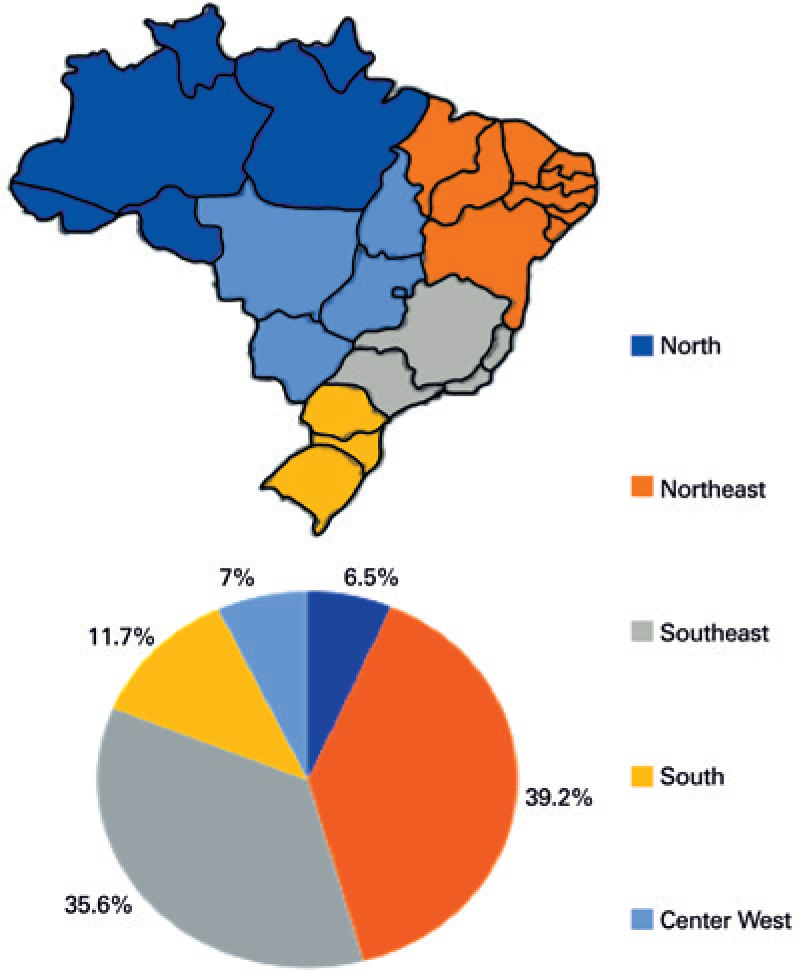
Distribution of hospital admissions at the Public Health System due to diagnosis of penile cancer in the Brazilian geographic regions, between 1992 and 2017

According to DATASUS, there were 9,592 hospital admissions related to PC from 1992 to 2017, with a mean of 365 admissions/year. The Northeast Region accounted for most admissions (3,757; 39.2%), followed by Southeast Region (3,416; 35.6%). In the North Region, 611 (0.7%) PC-related admissions were reported across the studied years (Figure 1).

**Figure 2:**
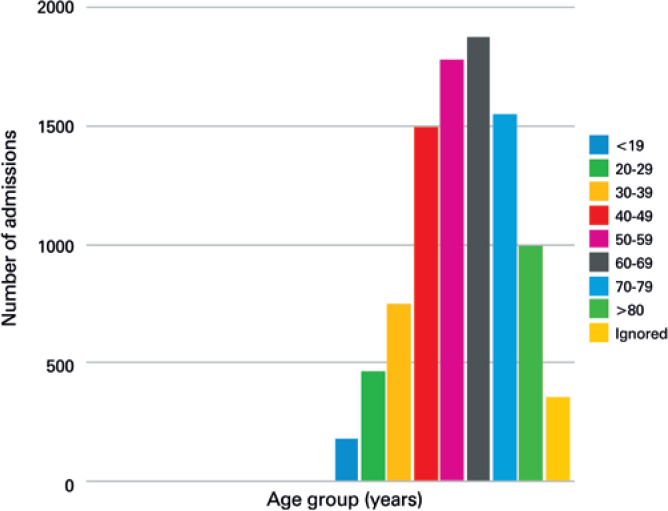
Age-stratified distribution of penile-cancer-related admissions in Brazil, from 1992-2017

There was a significant reduction in the absolute PC-related admissions per year in 2017, as compared to 1992. Penile cancer-related admissions accounted for 2.7/100,000 admissions and 1.7/100,000 admissions in Brazilian public hospitals, in 1992 and 2017, respectively, which represents a drop by 36% (p<0.001) (Table 1). When considering demographic data from each Brazilian region in 2017, there were 0.078 admission/100,000 people in the North Region, and 0.033 admission/100,000 people in the South Region, which is more economically developed. When stratified by age group, most admissions were of men between 50 and 69 years (Figure 2).

**Table 1:** Admissions due to penile cancer from 1992 to 2017 in Brazilian public hospitals

SUS admissions	1992	2017	p value
Penile cancer	406	203	
Total hospital admissions	14,583,191	11,468,707	
	0.0027%	0.0017%	p<0.02

SUS: *Sistema Único de Saúde*.

**Figure 3:**
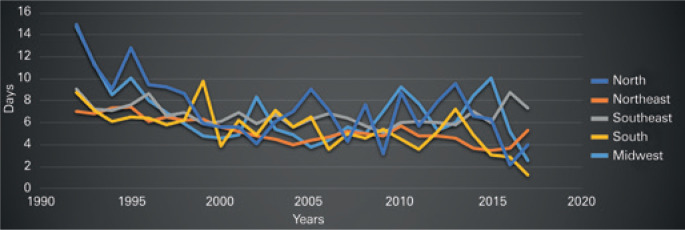
Hospital length of stay due to diagnosis of penile cancer per Brazilian geographic regions, Public Health System, 1992 to 2017

The mean hospital length of stay (LOS) was 6 days, with the longest stay in the Midwest Region. Patients seen in the Northeast Region were discharged earlier, with a mean hospital LOS of 5.3 days (Figure 3). It is worth mentioning that, in 1992, the hospital LOS achieved the maximum (8.6 days), and, in 2004, the minimum value (5 days).

The total expenses with PC-related admissions in this period was US$ 3,002,705.73, with an annual mean of US$ 115,488.68. Approximately 38% of the total amount was spent in Northeast Region, followed by Southeast (36%), South (12%), Midwest (7%) and North (5%) Regions.

The economic burden of PC in the North Region was US$ 1,143,334.05, from 1992 to 2017. The cost for each admission, in 2017, was US$ 221.02, totaling up to US$ 47,078.66 in expenses in that year. Across the studied years, PC-related expenses showed a drop by 77.2%. In 1992, PC costed SUS a total of US$ 193,502.05, accounting for 0.0046% of the total amount spent on the entire system that year. In 2017, PC-related costs reduced to 0.0010% with a total of US$ 47,078.66 (p<0.02, Table 2). The average amount spent per admission for North, Northeast, Southeast, South and Midwest regions was US$ 275.32; US$ 307.51; US$ 328.19; US$ 336.88 and US$ 318.10, respectively.

**Table 2:** Ratio of costs for treatment of penis cancer and total SUS costs in 1992 and 2017 in Brazilian public hospitals

Admisions	1992	2017	p value
Costs PC (US$)	193,502.05	47,078.66	
Total SUS costs (US$)	4,131,966,723.31	4,410,120,022.78	
	0.0046%	0.0010%	p<0.02

SUS: *Sistema Único de Saúde*; PC: penile cancer.

**Figure 4:**
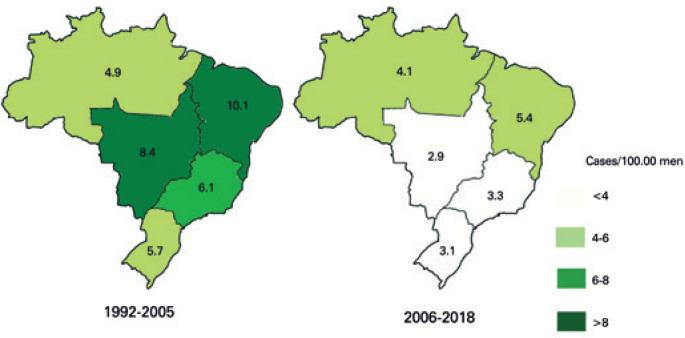
Incidence of admissions due to penile cancer per 100,000 men according to Brazilian regions from 1992-2005 and 2006-2018

According to INCA, the incidence of PC was 0.43/100,000 male Brazilian, in 2017, with the highest incidence rate in the Northeast Region. From 1992 to 2017, PC mortality rate in Brazil was 0.38/100,000 man, being 0.50/100,000 man in the North Region (Table 3). Mortality rates increased with age of diagnosis, being 0.07/100,000 among 30-39 years-old men, and reaching 2.02/100,000 for octogenarians. This increase in mortality rate with age was also noted when considering each region separately (Table 3). The incidence of hospital admission due to PC also decreased during the studied period (Figure 4).

**Table 3:** Penile cancer mortality rates per 100,000 males in Brazilian public hospitals, from 1992 to 2016, per Brazilian geographic regions and age group

Regions	Age group (years)								
	20-29	30-39	40-49	50-59	60-69	70-79	≥80	Ignored	Total
Midwest	0.02	0.19	0.46	0.86	1.78	2.84	6.07	2.19	0.47
Northeast	0.03	0.20	0.49	0.90	1.52	2.46	5.82	3.86	0.45
North	0.03	0.20	0.50	0.98	1.65	2.83	7.03	0.12	0.50
Southeast	0.02	0.12	0.35	0,66	1.14	2.03	4.42	1.73	0.34
South	0.02	0.10	0.25	0.54	1.23	2.31	5.44	0.00	0.34
	0.01	0.07	0.19	0.35	0.61	1.00	2.02	0.56	

Data from the Brazilian National Institute of Cancer.

## DISCUSSION

The data collected in our study brings some relevant findings. First, in the last 25 years, admissions related to PC decreased significantly, roughly by 50% (p<0.001). Considering that PC risk factors are strongly associated with lower socioeconomic status and poor hygiene, this decrease in admissions may reflect an improvement in social status and health education. Indeed, in 2009, the Brazilian Ministry of Health implemented a series of efforts aiming to promote male health through the National Policy for Comprehensive Health Care for Men (*Política Nacional de Atenção Integral à Saúde do Homem)*.^(^^21^^)^ Some studies reported the incidence of PC varied according to aspects of personal hygiene and religious practices. It is believed that the tumor develops through chronic irritative effects of smegma, a byproduct of bacterial action on the flaked cells within the preputial sac. Smegma has been implicated as a carcinogenic agent and its association with the development of PC has been widely observed.^(^^13^^)^ Other studies also report these factors as the initial cause of PC, including one by Frisch et al., which described three major risk factors: phimosis/long foreskin, low socioeconomic status and poor hygiene.^(^^22^^)^ These factors coincide with our results, in which higher incidences were observed in regions with lower Human Development Index (HDI).^(^^23^^)^ Consequently, personal hygiene and circumcision act as protective factors against PC. Maden et al., demonstrated the incidence of PC is lower when circumcision is performed in neonatal period and early childhood.^(^^24^^)^ It is reasonable to think that circumcision plays a role on decreasing the incidence of PC, improving glans exposure and hygiene. This effect was highlighted on a Danish population-based study reporting a decreasing incidence of PC from 1943 to 1990, in a population with less than 2% of men were circumcised before age 15 years. The same study stated that better hygiene may have an important impact on this decrease, since there was an incremental increase in the number of Danish households with baths (35% in 1940 to 90% in 1990).^(^^22^^)^

Second, as expected, our study revealed that most of admissions due to PC occurred in the Northeast region, followed by the Southeast Region. According to IBGE data, the Northeast region comprehends several places with the worst HDI in Brazil, having almost one-third of its populations based in rural areas.^(^^18^^,^^20^^,^^23^^)^ These characteristics may result on higher PC incidence and admission rates, due to lack of access to health and education services, along with poor hygiene. On the other hand, the Southeast region has a higher HDI and better access to health services, and its elevated number of PC admissions might be related to patient migration from underdeveloped areas from the Northeast to Southeast region, as previously suggested by Favorito et al. These authors found higher PC incidence in Northeast and Southeast regions, remarkably in the states of Maranhão and São Paulo.^(^^16^^)^ The first due to a large number of cases, and the second, for migration. It is our belief that the higher incidence of PC found on higher HDI areas may be attributed to the fact that patients often migrate to more developed areas of the country, seeking for diagnosis and treatment.^(^^15^^)^

Third, the costs related to PC management also showed a decrease by 72%, from 1992 to 2017 (p<0.02). The total amount that the Brazilian government allocated to fund the SUS remained stable across the years, and the amount spent on PC-related admission decreased in 2017. This fact might reflect that fewer new cases of PC are emerging, or that the cases are being managed in earlier stages, demanding fewer resources. However, it should be considered that these data do not include the outpatient treatment costs of these patients, such as visits, laboratory tests, imaging tests, and medication. It is also important to observe that our study does not report expenses with chemotherapy and lymphadenectomy for PC.

Based on our source of data and cost estimation methods, it is difficult to make comparisons between our findings and other published results. In our study, the average value of each admission ranged from US$ 275.32 to US$ 336.88, whereas in a study analyzing patients treated through private healthcare insurance, the mean expenditure on admission was US$ 25,948.00.^(^^25^^)^

Comparing total expenditures of admissions in SUS, in relation to urological tumors in 2018, testicular and penile neoplasms accounted for an expense of US$ 1.92 million (4.5% of total urogenital neoplasms). On the other hand, prostate cancer cost was U$ 27.1 million (61% of the all urologic malignancies).^(^^18^^)^ Another cost we could not measure in our study was absenteeism rates; besides the mean length of stay of 5.3 days, the social aspects and economic impact of PC might be even more significant, and are not analyzed in this study.

Lastly, our findings corroborate the current literature when it comes to higher mortality rates with increased age, especially among octogenarians. Across the years, PC mortality rates in Brazil presented a slight increase, which can be a result of health negligence and delay in obtaining medical specialized care.^(^^16^^)^ A significant number of cases and deaths by PC were observed in young adults, which leads to mutilation and death among sexually active men, similar to other national studies.^(^^16^^,^^17^^)^

Due to its epidemiologic design, our study does not allow definite conclusions regarding PC economic burden. The costs presented are underestimated. Our analysis was based on admission costs. Unfortunately we were not able to evaluate additional costs associated with lymphadenectomy, chemotherapy, as well as the secondary costs with complications after the initial treatment. Moreover, we could not evaluate the burden with outpatient care and social support. Yet, DATASUS does not include data from private healthcare system, only from the public system, so it did not fully measure the incidence and costs in the entire country. Despite the limitations exposed and considering the paucity of data on PC economic burden to Brazilian SUS, our data presents itself as an important tool to help developing government programs and health policies, since it outlines PC epidemiologic trends and admissions costs through the last quarter of a century in Brazil.

## CONCLUSION

Penile cancer is a disease with elevated mortality rates in regions with low human development index. Despite the decrease in penile-cancer-related admissions, the condition still causes a significant economic and social burden to Brazilian population and the Public Health System. As some studies have shown, some Brazilian regions might have the highest penile cancer incidence rates in the world, and further studies are needed, especially in regards to penile cancer outpatient costs and absenteeism, so as to understand the real impact of penile cancer in our population, and focus efforts on controlling such an aggressive and mutilating disease.

## References

[1] 1. Pettaway CA, Lynch Jr D, Davis D. Tumors of the penis. In: Wein AJ, Kavoussi LR, Novick AC. Campbell-Walsh Urology 9th ed. rev. Philadelphia: Saunders; 2007. p. 959-92.

[2] 2. Richter S, Ruether JD, Wood L, Canil C, Moretto P, Venner P, et al. Management of carcinoma of the penis: consensus statement from the Canadian Association of Genitourinary Medical Oncologists (CAGMO). Can Urol Assoc J. 2013;7(11-12):E797-811. 10.5489/cuaj.1794 PMC3879728 24475001

[3] 3. International Agency for Research on Cancer (IARC). Cancer incidence in five continents. Volume X [Internet]. Lyon (FR) IARC; 2014 [cited 2019 Apr 20]. Available from: http://ci5.iarc.fr/CI5I-X/old/vol10/CI5vol10.pdf

[4] 4. Barnholtz-Sloan JS, Maldonado JL, Pow-sang J, Giuliano AR. Incidence trends in primary malignant penile cancer. Urol Oncol. 2007;25(5):361-7. Erratum in: Urol Oncol. 2008;26(1):112. Guiliano, Ann R [corrected to Giuliano, Anna R]. 10.1016/j.urolonc.2006.08.029 17826651

[5] 5. Horenblas S. Lymphadenectomy for squamous cell carcinoma of the penis. Part 2: the role and technique of lymph node dissection. BJU Int. 2001;88(5):473-83. Review. 10.1046/j.1464-410x.2001.00379.x 11589660

[6] 6. Hernandez BY, Barnholtz-Sloan J, German RR, Giuliano A, Goodman MT, King JB, et al. Burden of invasive squamous cell carcinoma of the penis in the United States, 1998-2003. Cancer. 2008;113(10 Suppl):2883-91. 10.1002/cncr.23743 PMC2693711 18980292

[7] 7. Bleeker MC, Heideman DA, Snijders PJ, Horenblas S, Dillner J, Meijer CJ. Penile cancer: epidemiology, pathogenesis and prevention. World J Urol. 2009; 27(2):141-50. Review. 10.1007/s00345-008-0302-z 18607597

[8] 8. Morris BJ, Gray RH, Castellsague X, Bosch FX, Halperin DT, Waskett JH, et al. The strong protective effect of circumcision against cancer of the penis. Adv Urol. 2011;2011:812368. 10.1155/2011/812368 PMC3113366 21687572

[9] 9. Pow-Sang MR, Ferreira U, Pow-Sang JM, Nardi AC, Destefano V. Epidemiology and natural history of penile cancer. Urology. 2010;76(2 Suppl 1):S2-6. Review. 10.1016/j.urology.2010.03.003 20691882

[10] 10. Shavit O, Roura E, Barchana M, Diaz M, Bornstein J. Burden of human papillomavirus infection and related diseases in Israel. Vaccine. 2013; 31(Suppl 8):I32-41. Review. 10.1016/j.vaccine.2013.05.108 24229717

[11] 11. Dillner J, von Krogh G, Horenblas S, Meijer CJ. Etiology of squamous cell carcinoma of the penis. Scand J Urol Nephrol Suppl. 2000;(205):189-93. Review. 10.1080/00365590050509913 11144896

[12] 12. Suffrin G, Huben R. Benign and malignant lesions of the penis. In: JY G. Adult and Pediatric Urology. 2nd ed. Chicago: Year Book Medical Publisher; 1991. p. 1643.

[13] 13. Misra S, Chaturvedi A, Misra NC. Penile carcinoma: a challenge for the developing world. Lancet Oncol. 2004;5(4):240-7. Review. 10.1016/S1470-2045(04)01427-5 15050955

[14] 14. Skeppner E, Andersson SO, Johansson JE, Windahl T. Initial symptoms and delay in patients with penile carcinoma. Scand J Urol Nephrol. 2012; 46(5):319-25. 10.3109/00365599.2012.677473 22989150

[15] 15. Couto TC, Arruda RM, Couto MC, Barros FD. Epidemiological study of penile cancer in Pernambuco: experience of two reference centers. Int Braz J Urol. 2014;40(6):738-44. 10.1590/S1677-5538.IBJU.2014.06.04 25615242

[16] 16. Favorito LA, Nardi AC, Ronalsa M, Zequi SC, Sampaio FJ, Glina S. Epidemiologic study of penile câncer in Brazil. Int Braz J Urol. 2018;34(5):587-91. discussion 591-3. 10.1590/s1677-55382008000500007 18986562

[17] 17. Coelho RW, Pinho JD, Moreno JS, Garbis DV, do Nascimento AM, Larges JS, et al. Penile cancer in Maranhão, Northeast Brazil: the highest incidence globally? BMC Urol. 2018;18(1):50. 10.1186/s12894-018-0365-0 PMC5975591 29843769

[18] 18. Brasil. Ministério da Saúde. Departamento de Informôtica do SUS (DATASUS). Informações de Saúde (TABNET). Assistência a Saúde [Internet]. Brasília (DF): DATASUS; 2019 [citado 2019 Abr 20]. Disponível em: http://www2.datasus.gov.br/DATASUS/index.php?area=0202

[19] 19. Brasil. Ministério da Saúde. Instituto Nacional de Câncer José Alencar Gomes da Silva (INCA). Informações do Registro de Câncer de Base Populacional [Internet]. INCA; 2019 [citado 2019 Mar 20]. Disponível em: https://www.inca.gov.br/BasePopIncidencias/Home.action

[20] 20. Instituto Brasileiro de Geografia e Estatística (IBGE). Ministério do Planejamento, Orçamento e Gestão. Projeção da população do Brasil e das Unidades da Federação por sexo e idade: 2010-2060 [Internet]. Governo Federal do Brasil; 2010 [citado 2019 Abr 20]. Disponível em: https://www.ibge.gov.br/estatisticas/sociais/populacao/9109-projecao-da-populacao.html?=&t=resultados

[21] 21. Brasil. Ministério da Saúde. Gabinete de Ministro. Portaria n. 1.944, de 27 de agosto de 2009. Institui no âmbito do Sistema Único de Saúde (SUS), a Política Nacional de Atenção Integral à Saúde do Homem [Internet]. Brasília (DF): Diôrio Oficial da União; 2009 [citado 2019 Abr 10]. Disponível em: http://bvsms.saude.gov.br/bvs/saudelegis/gm/2009/prt1944_27_08_2009.html

[22] 22. Frisch M, Friis S, Kjaer SK, Melbye M. Falling incidence of penis cancer in an uncircumcised population (Denmark 1943-90). BMJ. 1995;311(7018):1471. 10.1136/bmj.311.7018.1471 PMC2543732 8520335

[23] 23. Instituto de Pesquisa Econômica Aplicada (IPEA). Radar IDHM: evolução do IDHM e de seus índices componentes no período de 2012 a 2017 [Internet]. Brasília (DF): IPEA; 2019 [citado 2019 Abr 18]. Disponível em: http://www.ipea.gov.br/portal/images/stories/PDFs/livros/livros/190416_rada_IDHM.pdf

[24] 24. Maden C, Sherman KJ, Beckmann AM, Hislop TG, Teh CZ, Ashley RL, et al. History of circumcision, medical conditions, and sexual activity and risk of penile cancer. J Natl Cancer Inst. 1993;85(1):19-24. 10.1093/jnci/85.1.19 8380060

[25] 25. Lairson DR, Wu CF, Chan W, Fu S, Hoffman KE, Pettaway CA. Mean treatment cost of incident cases of penile cancer for privately insured patients in the United States. Urol Oncol. 2019;37(4):294.e17-294.e25. 10.1016/j.urolonc.2019.01.004 30660492

