# Supplementary material for: Penile cancer trends and economic burden in the Brazilian public health system
Source: Einstein (Sao Paulo). 2020 Oct 29;18:eAO5577. doi: 10.31744/einstein_journal/2020AO5577 (PMC7607917; doi:10.31744/einstein_journal/2020AO5577)
Supplement: Translation

# Translation: Tendências e carga econômica do câncer de pênis no sistema público de saúde brasileiro

**Authors:** Fernando Korkes, Antônio Flávio Silva Rodrigues, Willy Baccaglini, Frederico Timóteo Silva Cunha, Júlio Slongo, Philippe Spiess, Sidney Glina

**Affiliations:** 1 Santo AndréSP Brasil Faculdade de Medicina do ABC, Santo André, SP, Brasil.; 2 Tampa United States University of South Florida, Tampa, United States.; 3 Tampa United States H. Lee Moffitt Cancer Center & Research Institute, Tampa, United States.

**Keywords:** Neoplasias penianas, Custos e anôlise de custo, Carcinoma de células escamosas, Indicadores de desenvolvimento, Saúde pública, Sistema Único de Saúde

## Abstract

**Objetivo:**

Reunir informações sobre as tendências epidemiológicas do câncer de pênis e seu impacto econômico no Sistema Único de Saúde nos últimos 25 anos.

**Métodos:**

O banco de dados de informações do Sistema Único de Saúde foi utilizado como fonte primária de dados de janeiro 1992 a dezembro 2017. Os dados demortalidade e incidência do Instituto Nacional de Câncer José Alencar Gomes da Silva foram coletados usando a Classificação Internacional de Doença CID10 C60. Os dados demográficos da população brasileira foram obtidos do último censo do Instituto Brasileiro de Geografia e Estatística, realizado em 2010, e em sua revisão, de 2017.

**Resultados:**

Ocorreram 9.743 internações relacionadas ao câncer de pênis de 1992 a 2017. Houve redução (36%) nas internações anuais absolutas em 2017 em comparação com 1992 (2,7 *versus* 1,7 por 100.000; p<0,001). Os gastos com internações neste período foram de US$ 3,002,705.73 (US$ 115,488.68/ano). Cerca de 38% do valor total foi gasto na Região Nordeste. Em 1992, o câncer de pênis custou US$ 193,502.05 ao sistema público, enquanto em 2017 reduziu para US$ 47,078.66 (p<0,02). A incidência em 2017 foi de 0,43/100.000 brasileiro do sexo masculino, com a maior taxa de incidência encontrada na Região Nordeste. De 1992 a 2017, as taxas de mortalidade por câncer de pênis foram de 0,38/100.000 homem, sendo 0,50/100.000 homem na Região Norte.

**Conclusão:**

Apesar da diminuição nas hospitalizações, o câncer de pênis ainda impõe uma carga econômica e social significativa à população brasileira e ao Sistema Único de Saúde.

## INTRODUÇÃO

O câncer de pênis (CP) é uma doença rara, que representa de 0,4% a 0,6% de todas as neoplasias malignas entre homens nos Estados Unidos e na Europa.^(^^1^^,^^2^^)^ No entanto, está bem documentado que a incidência de CP é maior nos países desenvolvidos, podendo ser responsável por até 10% a 20% dos tumores urogenitais masculinos nessas regiões.^(^^3^^,^^4^^)^ Embora relativamente raro, o CP tem taxa de sobrevida em 5 anos de cerca de 50% (mais de 85% para pacientes com linfonodos negativos, e de 29% a 40% quando positivos) e, muitas vezes, leva à desfiguração devastadora.^(^^5^^)^

Em geral, o CP apresenta-se como uma lesão palpável, visível e indolor no pênis. Não obstante, os pacientes podem se queixar de dor, corrimento, sangramento ou mal odor, especialmente se houver demora em procurar tratamento médico.^(^^6^^)^ Aproximadamente 95% dos CP originam nas células epiteliais escamosas e podem ser classificados como carcinoma de células escamosas (CCE) ou neoplasia intraepitelial peniana.^(^^7^^)^ A capacidade de realizar um bom exame físico tem papel primordial no diagnóstico precoce do CCE peniano, e a circuncisão reduz a incidência de tumores.^(^^8^^)^ Essa hipótese é exemplificada pelos países em que o sistema médico e as práticas religiosas resultam em um maior índice de circuncisões. Israel, por exemplo, tem a menor incidência de CP no mundo (0,1 caso em 100 mil homens).^(^^9^^,^^10^^)^ Por outro lado, homens com fimose chegam a ter risco 60% maior de apresentar CP.^(^^11^^,^^12^^)^ Em última análise, é amplamente reconhecido que piores condições socioeconômicas estão relacionadas a uma maior ocorrência do CP. Um estudo de base populacional, realizado na Suécia, relatou risco aumentado de CP invasivo entre os indivíduos com menor renda e de menor nível educacional. Provavelmente isso se relaciona a alguns fatores, como a demora em buscar atendimento médico, o estigma da doença, o medo do tratamento e a falta de conhecimento sobre o diagnóstico.^(^^13^^,^^14^^)^

No Brasil, o CP pode ser responsável por 2,1% de todas as neoplasias em homens e afeta principalmente os habitantes das Regiões Norte e Nordeste,^(^^15^^,^^16^^)^ duas ôreas geográficas historicamente marcadas por grande desigualdade social e extrema pobreza. Um recente estudo de coorte retrospectivo de centro único mostrou taxa de incidência ajustada por idade que chega a 6,1/100 mil casos entre os habitantes do sexo masculino do estado do Maranhão, sugerindo que esse estado da Região Nordeste pode ter a mais elevada taxa de incidência de CP no mundo.^(^^17^^)^ Embora o Brasil se destaque entre os países com maior incidência de CP no mundo, não há dados atualizados confiáveis referentes ao impacto econômico da doença sobre o Sistema Único de Saúde (SUS).

## OBJETIVO

Reunir informações sobre as tendências epidemiológicas do câncer de pênis e seu impacto econômico no sistema público de saúde brasileiro ao longo dos últimos 25 anos.

## MÉTODOS

O Departamento de Informática do Sistema Único de Saúde (DATASUS) representa o principal empreendimento do governo federal brasileiro na coleta de dados do SUS e foi usado como fonte primária de dados para nosso estudo, juntamente do Sistema de Informações Hospitalares (SIH) do SUS.^(^^18^^)^ Foram analisados dados epidemiológicos de janeiro de 1992 a dezembro de 2017, com informações compiladas das internações em hospitais públicos registradas sob os seguintes códigos: amputação do pênis, amputação oncológica do pênis e amputação peniana total estendida. Os gastos de saúde pública com o tratamento do CP foram estimados usando-se o código de amputação peniana e analisados de acordo com a inflação anual em dólares americanos. Infelizmente, não pudemos quantificar outros gastos com exames pré-operatórios, linfadenectomias, complicações a longo prazo ou reinternações. Esse banco de dados inclui informações de todos os hospitais públicos do país, que oferecem atendimento médico a cerca de 170 milhões de brasileiros.

Os dados de mortalidade e incidência do Instituto Nacional de Câncer José de Alencar Gomes da Silva (INCA) foram coletados utilizando a Classificação Internacional de Doenças (CID-10) C60.^(^^19^^)^ Os dados demográficos da população brasileira durante o período estudado foram obtidos do último recenseamento do Instituto Brasileiro de Geografia e Estatística (IBGE), realizado em 2010, e sua revisão, de 2017.^(^^20^^)^

A análise estatística foi realizada usando o (SPSS), versão 13.0 (SPSS para Mac OS X, SPSS, Inc., Chicago, Illinois). Os grupos foram comparados quanto às diferenças entre 1992 e 2017 pelo teste χ^2^ de Pearson, e a significância estatística foi determinada em p<0,05.

## RESULTADOS

Segundo o DATASUS, houve 9.592 internações por CP de 1992 a 2017, com média de 365 internações por ano. A Região Nordeste foi responsável pela maioria das internações (3.757; 39,2%), seguida pela Sudeste (3.416; 35,6%). Na Região Norte, 611 (0,7%) internações por CP foram relatadas ao longo dos anos estudados (Figura 1).

**Figura 1:** Distribuição das internações no Sistema Único de Saúde por diagnóstico de câncer de pênis, nas regiões geográficas do Brasil, de 1992 a 2017

Houve redução significativa do número absoluto de internações anuais por CP em 2017, em comparação com 1992. Em 1992, as internações por CP representaram 2,7/100 mil internações nos hospitais públicos brasileiros, ao passo que, em 2017, esse número foi reduzido a 1,7/100 mil internações, o que representa decréscimo de 36% (p<0,001) (Tabela 1). Levando em considerações os dados demográficos de cada região brasileira em 2017, houve 0,078 internação/100 mil pessoas na Região Norte, ao passo que, na Região Sul (mais desenvolvida economicamente), ocorreram 0,033 internação por 100 mil pessoas. Ao serem estratificadas por faixa etária, a maioria das internações foi de homens entre 50 e 69 anos de idade (Figura 2).

**Tabela 1:** Relação de internações por câncer de pênis e total de admissões hospitalares em 1992 e 2017

Hospitalizações pelo SUS	1992	2017	Valor de p
Câncer de pênis	406	203	
Total de admissões hospitalares	14.583.191	11.468.707	
	0,0027%	0,0017%	p<0,02

SUS: Sistema Único de Saúde.

**Figura 2:** Distribuição com estratificação etária das internações por câncer de pênis no Brasil, de 1992 a 2017

O tempo médio de permanência hospitalar (TPH) foi de 6 dias, com o maior período de internação ocorrendo na Região Centro-Oeste. Os pacientes cuja doença foi tratada na Região Nordeste tiveram alta hospitalar mais cedo, com tempo médio de hospitalização de 5,3 dias (Figura 3). É interessante notar que, em 1992, o TPH atingiu o máximo (8,6 dias) e, em 2004, atingiu o mínimo (5 dias).

**Figura 3:** Tempo de internação devido ao diagnóstico de câncer de pênis, por regiões geográficas brasileiras, no Sistema Único de Saúde, de 1992 a 2017

O total de gastos com internações por CP nesse período foi de US$ 3,002,705.73, com média anual de US$ 115,488.68. Cerca de 38% do valor total foi gasto na Região Nordeste, seguida pelas Regiões Sudeste (36%), Sul (12%), Centro-Oeste (7%) e Norte (5%).

A carga econômica do CP na Região Norte foi de US$ 1,143,334.05 de 1992 a 2017. O custo por internação, em 2017, foi US$ 221.02, totalizando US$ 47,078.66 em despesas naquele ano. Ao longo dos anos estudados, as despesas por CP tiveram decréscimo de 77,2%. Em 1992, o CP custou ao SUS US$ 193,502.05, sendo responsável por 0,0046% do total gasto em todo o sistema naquele ano. Em 2017, os gastos referentes ao CP reduziram para 0,0010%, com total de US$ 47,078.66 (p<0,02, Tabela 2). O valor médio gasto por internação nas Regiões Norte, Nordeste, Sudeste, Sul e Centro-Oeste foram, respectivamente, US$ 275.32; US$ 307.51; US$ 328.19; US$ 336.88 e US$ 318.10.

**Tabela 2:** Relação de gastos por tratamento de câncer de pênis e gastos totais do SUS em 1992 e 2017

Hospitalizações	1992	2017	Valor de p
Gastos com CP (US$)	193,502.05	47,078.66	
Total de gastos do SUS (US$)	4,131,966,723.31	4,410,120,022.78	
	0,0046%	0,0010%	p<0,02

SUS: Sistema Único de Saúde; CP: câncer de pênis.

Segundo o INCA, a incidência de CP foi de 0,43/100 mil homem brasileiro, em 2017, com a maior taxa de incidência na Região Nordeste. De 1992 a 2017, a taxa de mortalidade por CP no Brasil foi 0,38/100 mil homem, sendo 0,50/100 mil homem na região Norte (Tabela 3). As taxas de mortalidade aumentaram com a idade do diagnóstico, sendo de 0,07/100 mil entre os homens de 30 a 39 anos, chegando a 2,02 por 100 mil entre os octogenários. Esse aumento na taxa de mortalidade com a idade também foi notado ao se levar em consideração cada região separadamente (Tabela 3). A incidência de internações hospitalares por CP também diminuiu no período estudado (Figura 4).

**Tabela 3:** Taxas de mortalidade do câncer de pênis por 100 mil homens nos hospitais públicos brasileiros de 1992 a 2016, segundo regiões geográficas brasileiras e faixa etária

Regiões	Faixa etária (anos)								
	20-29	30-39	40-49	50-59	60-69	70-79	≥80	Ignorada	Total
Centro-Oeste	0,02	0,19	0,46	0,86	1,78	2,84	6,07	2,19	0,47
Nordeste	0,03	0,20	0,49	0,90	1,52	2,46	5,82	3,86	0,45
Norte	0,03	0,20	0,50	0,98	1,65	2,83	7,03	0,12	0,50
Sudeste	0,02	0,12	0,35	0,66	1,14	2,03	4,42	1,73	0,34
Sul	0,02	0,10	0,25	0,54	1,23	2,31	5,44	0,00	0,34
	0,01	0,07	0,19	0,35	0,61	1,00	2,02	0,56	

Dados do Instituto Nacional de Câncer.

**Figura 4:** Incidência de internações por câncer de pênis por 100 mil homens, segundo regiões brasileiras, de 1992 a 2005, e de 2006 a 2016

## DISCUSSÃO

Os dados coletados em nosso estudo trazem alguns achados relevantes. Primeiro, nos últimos 25 anos, as internações relacionadas ao CP diminuíram significativamente, em torno de 50% (p<0,001). Considerando que os fatores de risco para CP estão fortemente associados às baixas condições socioeconômico e de higiene, essa diminuição nas hospitalizações pode refletir uma melhora nas condições sociais e na educação em saúde. De fato, em 2009, o Ministério da Saúde brasileiro implementou uma série de esforços com o objetivo de promover a melhoria da saúde masculina, por meio da Política Nacional de Atenção Integral à Saúde do Homem.^(^^21^^)^ Alguns estudos relatam que a incidência de CP varia de acordo com aspectos de higiene pessoal, bem como práticas religiosas. Acredita-se que o tumor se desenvolva por meio de efeitos irritativos crônicos do esmegma, um subproduto da ação bacteriana nas células descamadas do saco prepucial. O esmegma tem sido implicado como agente cancerígeno, e sua associação com o desenvolvimento da CP é amplamente observada.^(^^13^^)^ Outros estudos também relatam esses fatores como causa inicial do CP, como Frisch et al., que descreveram três principais fatores de risco: fimose/prepúcio longo, baixo *status* socioeconômico e higiene precôria.^(^^22^^)^ Esses fatores coincidem com nossos resultados, nos quais foram observadas incidências mais altas em regiões com menor Índice de Desenvolvimento Humano (IDH).^(^^23^^)^ Consequentemente, a higiene pessoal e a circuncisão atuam como fatores de proteção contra o CP. Maden et al., demonstraram que a incidência de CP é menor quando a circuncisão é realizada no período neonatal e na primeira infância.^(^^24^^)^ É razoável supor que a circuncisão desempenha papel na diminuição da incidência de CP, melhorando a exposição e a higiene da glande. Esse efeito foi destacado em um estudo populacional dinamarquês, que encontrou incidência decrescente de CP de 1943 a 1990 em uma população em que menos de 2% dos homens foram circuncidados antes dos 15 anos. O mesmo estudo afirmou que uma melhor higiene pode ter impacto importante nessa diminuição, pois houve mudança incremental no número de casas dinamarquesas com banho (de 35%, em 1940, para 90%, em 1990).^(^^22^^)^

Segundo, conforme esperado, nosso estudo revelou que a maioria das internações por CP ocorreu na Região Nordeste, seguida pela Região Sudeste. De acordo com dados do IBGE, a Região Nordeste compreende vários locais com o pior IDH do Brasil, tendo quase um terço de sua população em áreas rurais.^(^^18^^,^^20^^,^^23^^)^ Essas características podem resultar em maior incidência de CP e maiores taxas de hospitalização, devido à falta de acesso a serviços de saúde e educação, além da falta de higiene. Por outro lado, a Região Sudeste possui um IDH mais alto e melhor acesso a serviços de saúde, e seu elevado número de internações por CP pode estar relacionado à migração de pacientes de ôreas subdesenvolvidas do Nordeste para o Sudeste, conforme sugerido anteriormente por Favorito et al. Esses autores encontraram maior incidência de CP nas Regiões Nordeste e Sudeste, notadamente nos estados do Maranhão e São Paulo.^(^^16^^)^ A primeira se deu devido ao grande número de casos, e a segunda, à migração. É nossa convicção que a maior incidência de CP encontrada em áreas com maior IDH pode ser atribuída ao fato de os pacientes migrarem para áreas mais desenvolvidas do país, buscando diagnóstico e tratamento.^(^^15^^)^

Terceiro, os custos relativos ao tratamento de CP também mostraram redução de 72%, de 1992 a 2017 (p<0,02). O valor total que o governo brasileiro alocou para financiar o SUS permaneceu estável ao longo dos anos, e o valor gasto em hospitalização por CP diminuiu em 2017. Esse cenário pode refletir o fato de que menos casos novos de CP estão surgindo, ou que os casos estão sendo gerenciados em estágios mais iniciais, exigindo menos recursos. No entanto, deve-se ponderar que esses dados não incluem os custos de tratamento ambulatorial desses pacientes, como consultas, exames laboratoriais, exames de imagem e medicamentos. Também é importante notar que nosso estudo não relata gastos com quimioterapia e linfadenectomia por CP.

Com base em nossa fonte de dados e métodos de estimativa de custos, é difícil fazer comparações entre nossas descobertas e outros resultados publicados. Em nosso estudo, o valor médio de cada hospitalização variou de US$ 275.32 a US$ 336.88, ao passo que, em estudo que analisou pacientes tratados com seguro privado, o gasto médio em internação foi de US$ 25,948.00.^(^^25^^)^

Comparando os gastos totais com hospitalização no SUS, no tocante aos tumores urológicos em 2018, as neoplasias testiculares e penianas representaram despesa de US$ 1.92 milhão (4,5% do total das neoplasias urogenitais). Por outro lado, o custo do câncer de próstata foi de U$ 27.1 milhões (61% de todas as neoplasias urológicas).^(^^18^^)^ Outro custo que não pudemos medir em nosso estudo foram as taxas de absenteísmo do trabalho; além do período médio de internação de 5,3 dias, os aspectos sociais e o impacto econômico do CP podem ser ainda mais significativos e não foram analisados neste estudo.

Por fim, nossos achados corroboram a literatura atual, no tocante a taxas mais altas de mortalidade com o aumento da idade, principalmente entre os octogenôrios. Ao longo dos anos, as taxas de mortalidade por CP no Brasil apresentaram ligeiro aumento, que pode ser resultado de negligência na saúde e atraso na obtenção de cuidados médicos especializados.^(^^16^^)^ Um número significativo de casos e mortes por CP foi observado em adultos jovens, o que resulta em mutilação e morte entre homens sexualmente ativos, semelhante a outros estudos nacionais.^(^^16^^,^^17^^)^

Devido ao seu desenho epidemiológico, nosso estudo não permite conclusões definitivas sobre a carga econômica do CP. Os custos apresentados estão subestimados. Nossa análise foi baseada nos custos de hospitalização. Infelizmente, não fomos capazes de avaliar os custos adicionais associados à linfadenectomia e à quimioterapia, bem como custos secundôrios com complicações após o tratamento inicial. Além disso, não foi possível avaliar os gastos com atendimento ambulatorial e apoio social. Ainda, o DATASUS não inclui dados do sistema de saúde privado (apenas do sistema público), e, portanto, não foram totalmente mensurados a incidência e os custos em todo o país. Apesar das limitações expostas e levando em consideração a escassez de dados sobre o impacto econômico do CP para o SUS brasileiro, nossos dados apresentam-se como ferramenta importante para ajudar no desenvolvimento de programas e políticas de saúde do governo, ao traçarem tendências epidemiológicas e custos de hospitalizações por CP no último quarto de século no Brasil.

## CONCLUSÃO

O câncer de pênis é uma doença com elevada taxa de mortalidade em regiões com Índice de Desenvolvimento Humano baixo. Apesar do decréscimo no número de internações por câncer de pênis, ele ainda tem impacto econômico e social significativo para a população brasileira e seu sistema público de saúde. Conforme mostraram alguns estudos, hô regiões específicas do Brasil que têm a maior taxa de incidência de câncer de pênis do mundo, fazendo-se necessôrio que se realizem mais estudos, em especial no tocante aos custos do atendimento ambulatorial do câncer de pênis e às perdas de dias de trabalho, para entender o real impacto que o câncer de pênis tem sobre nossa população e concentrar esforços no controle dessa doença agressiva e mutiladora.
